# Distributional responses to climate change of two maple species in southern China

**DOI:** 10.1002/ece3.10490

**Published:** 2023-08-31

**Authors:** De Tuan Liu, Jian Ying Chen, Wei Bang Sun

**Affiliations:** ^1^ Yunnan Key Laboratory for Integrative Conservation of Plant Species with Extremely Small Populations Key Laboratory for Plant Diversity and Biogeography of East Asia Kunming Institute of Botany, Chinese Academy of Sciences Kunming China; ^2^ University of the Chinese Academy of Sciences Beijing China; ^3^ School of Life Sciences Yunnan University Kunming China; ^4^ Forest Seed and Seedling General Station of Yunnan Province Kunming China

**Keywords:** distribution modelling, global warming, the Hengduan Mountains, the Qinghai‐Tibetan Plateau

## Abstract

Climate change is a major factor affecting biodiversity and species distribution, particularly of montane species. Species may respond to climate change by shifting their range to higher elevations. The southeastern Qinghai‐Tibetan Plateau (QTP) and the Hengduan Mountains are considered as global biodiversity hotspots. However, information on the response of maple species to climate change in these regions was limited. Therefore, we selected two maple species that occur there and assessed changes in their habitat suitability under past, present and future climate scenarios in Biomod2. The results showed that temperature seasonality (bio4) was the most critical factor influencing their potential distributions. The distribution of potentially suitable habitat for *Acer caesium* and *Acer stachyophyllum* was predicted to be larger during the LGM compared to the present. Under the current climate scenario, the largest areas of potentially suitable habitat for these species were mainly located in southeastern Tibet, the Hengduan Mountains in northwestern Yunnan and western Sichuan, the Qinling‐Daba Mountains in southern Gansu and the Wumeng‐Daliang Mountains in northeastern Yunnan, western Guizhou and southeastern Sichuan. Under future climate change scenarios, the predicted loss of suitable habitat areas for these two species ranged from 13.78% to 45.71% and the increase ranged from 18.88% to 57.98%, with an overall increasing trend. The suitable habitat areas were predicted to shift towards the eastern parts of the QTP under both the pessimistic and optimistic future climate change scenarios in the 2050s and the 2070s, which became evident as global warming intensified, particularly in the eastern QTP and the Hengduan Mountains. Our results highlight the possibility that the diverse topography along altitudinal gradients in the QTP and the Hengduan Mountains may potentially mitigate the range contraction of mountain plants in response to climate warming. These findings provide a basis for planning conservation areas, planting and species conservation in the mountainous areas of southern China under the anticipated global warming.

## Graphical abstract

The potential distribution of *A*. *caesium* and *A*. *stachyophyllum* was projected using Biomod2. Temperature seasonality (bio4) was the most critical factor influencing the potential distribution of all two maple species. Suitable habitat areas for these two species would increase under future climate change scenarios. Range shifts of widespread species in response to climate change should be incorporated into conservation planning.

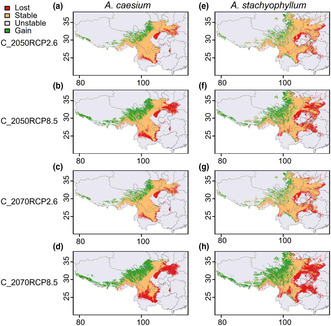

## 1 INTRODUCTION

Anthropogenic climate change has already had profound affects on species distributions and has resulted directly or indirectly in a huge loss of global biodiversity (Song et al., 2021). Global climate change is also likely to have a strong impact on ecosystem function, community structure, vegetation type and population genetic diversity in the future (Pio et al., 2014; Wang et al., 2021). If global warming reaches the levels predicted by the end of this century, roughly 20%–30% of plants and animals will be at risk of extinction (Penteriani et al., 2019). In response to climate change, species may shift their ranges, usually to higher altitudes or latitudes (Chhetri et al., 2018) or adapt to their changing environments (He et al., 2019). Montane species are more vulnerable and sensitive to climate change than species at lower elevations (Lenoir et al., 2008). Approximately 31%–51% of subalpine species and 19%–46% of montane species will lose more than 80% of their suitable habitat by 2070–2100 in the face of global change (Engler et al., 2011). In addition, montane species are often restricted to the summits of mountainous regions and often face a ‘nowhere to go’ scenario due to climate change (He et al., 2019). Therefore, modelling and predicting the response of montane species to climate change is, therefore, becoming increasingly important (Liang et al., 2018).

The Qinghai‐Tibetan Plateau (QTP), known as the ‘Roof of the World’ and the ‘Third Pole of the World’, is the highest plateau on Earth, with an average elevation of over 4000 m. It is also a crucial region for ecosystem and biodiversity conservation (Yu et al., 2019), with over ~12,000 native seed plants (Zhang et al., 2016). Due to the diverse environmental heterogeneity, the QTP is considered to have been an important refugium for *Aconitum gymnandrum* Maxim. (Wang et al., 2009) during the Last Glacial Maximum (LGM, 26.5–19 ka) (Clark et al., 2009), and is considered as the likely cradle of many temperate taxa in Eurasia, including mammals and plants (Yu et al., 2019). The QTP is also predicted to be a future climate refugium for *Meconopsis* species (Wang et al., 2021) and for cold coniferous forests in southwestern China (Dakhil et al., 2019) under global climate change. Several lineages have been inferred to have originated in the Central Asian and adjacent regions, and then migrated into the QTP, such as *Solms‐laubachia* Muschl. ex Diels (Yue et al., 2009) and *Incarvillea* Juss. (Chen et al., 2005; Rana et al., 2021). Species in the genus *Acer* are thought to have originated from the Hengduan Mountains in southeastern QTP, together with the provinces of Hubei, Hunan and eastern Sichuan, from where they subsequently spread to West Asia, Europe, North Africa, North America, the Malay Peninsula and Indonesia (Xu, 1996, 1998). Climate change in the past may have been a major driver for the migration of the genus *Acer* (Gao et al., 2020).

Species distribution modelling (SDM) can be used to study the historical, current and future distributions of species, providing valuable additional insights into species dynamics across time (Elith & Leathwick, 2009; Wang et al., 2015). To our knowledge, only a handful of studies have evaluated the response of maple species to climate change. The distribution of *Acer ginnala* Maxim. (Zhao et al., 2016), *Acer davidii* Franch. (Su et al., 2021) and *Acer cordatum* Pax (Liu, Sun et al., 2022) were predicted to move to the eastern parts of their current range in the future. The predicted high‐richness regions in the current conditions in southeastern Tibet of threatened maples were predicted to be greatly reduced in the future (Liu, Yang et al., 2022).

**FIGURE 1:**
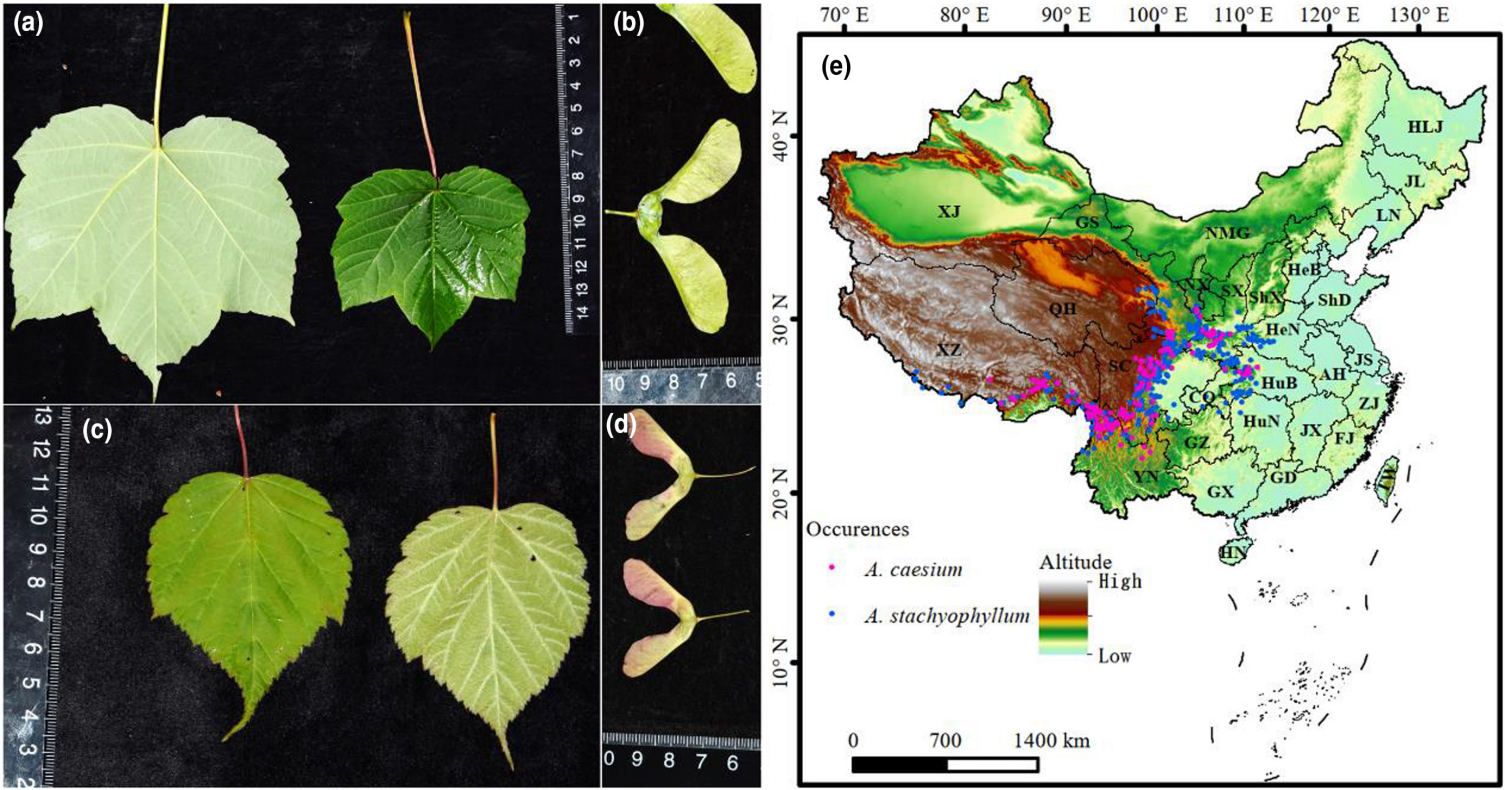
Photographs of *Acer caesium* (a, b) and *Acer stachyophyllum* (c, d) and their geographical distribution in China (e). (a, c) leaves; (b, d) samara; blue dots indicate occurrence records of *A*. *stachyophyllum* and purple dots, *A*. *caesium*. The Chinese Provinces are abbreviated as follows: AH, Anhui; CQ, Chongqing; FJ, Fujian; GD, Guangdong; GS, Gansu; GX, Guangxi; GZ, Guizhou; HeB, Hebei; HeN, Henan; HLJ, Heilongjiang; HN, Hainan; HuB, Hubei; HuN, Hunan; JL, Jilin; JS, Jiangsu; JX, Jiangxi; LN, Liaoning; NMG, Neimenggu; NX, Ningxia; QH, Qinghai; SC, Sichuan; ShD, Shandong; ShX, Shanxi; SX, Shaanxi; TW, Taiwan; XJ, Xinjiang; XZ, Xizang; YN, Yunnan; ZJ, Zhejiang.

*Acer caesium* Wall. ex Brandis (Figure 1a,b) and *Acer stachyophyllum* Hiern (Figure 1c,d), two maples of the genus *Acer*, are currently widespread in the montane regions of the QTP and the Hengduan Mountains (Xu et al., 2008) and there is a substantial collection of herbarium specimens available that can provide sufficient data for SDM. They prefer cool and humid climates and are sympatric in open montane forests over much of the elevation range 2300–3200 m (Xu et al., 2008). Changes in the distribution of plant species in response to climate warming are more complex than previously thought (Liang et al., 2018). And how these two species may respond to global change is largely unknown. To quantify the dynamics of changes in suitable habitat of *A*. *caesium* and *A*. *stachyophyllum* over different time periods, and to test whether they have a migration trend from the Hengduan Mountains to the QTP, we performed SDM analysis using presence data and bioclimatic variables. We assumed that the two maple species with current distribution in the QTP may have migrated there in the past, as Xu (1996) suggested that maple species originated in the Hengduan Mountains. We also hypothesized that these two montane maple species would experience a significant reduction in their suitable habitat range in the future, as montane species are more vulnerable to climate change (Engler et al., 2011; Lenoir et al., 2008).

## 2 MATERIALS AND METHODS

### 2.1 Occurrence data

We extracted occurrence data for each of the two maple species from online databases, including the Chinese Virtual Herbarium (CVH, https://www.cvh.ac.cn, accessed on 2020‐12‐27), the China National Specimen Information Infrastructure (NSII, http://www.nsii.org.cn/2017/home.php, accessed on 2021‐12‐24) and the Global Biodiversity Information Facility (GBIF, https://www.gbif.org, accessed on 2021‐12‐24). We further searched the Chinese Field Herbarium (CFH, http://www.cfh.ac.cn, accessed on 2021‐01‐10) and the Plant Photo Bank of China (PPBC, http://ppbc.iplant.cn, accessed on 2021‐01‐12) to obtain observation records. In addition, we also included records from our fieldwork. Totally, 561 and 2176 occurrence data of *A*. *caesium* and *A*. *stachyophyllum* were obtained, respectively.

Following previously published methods (Liu, Yang et al., 2022), we cleaned the occurrence data and removed duplicate records within a 5‐km range to lower potential autocorrelation through spatial filtering using the *spatially rarify occurrence data* function of SDMToolbox v2.4 (Brown, 2014) implemented in ArcGIS v10.2. Finally, 149 and 392 effective data records of *A*. *caesium* and *A*. *stachyophyllum* were obtained, respectively, and were used for further analysis. The geographical distribution of these two studied maple species in China is shown in Figure 1e.

### 2.2 Environmental variable

As the highest resolution of bioclimatic variables for the LGM period was only available at 2.5 arcminutes, to estimate the responses of these two maple species to climate change, 19 bioclimatic variables (bio1–bio19, Table S1) for current climatic conditions (average for 1960–1999) were downloaded from WorldClim v1.4 at a resolution of 2.5′ (0.044915°), about 5 km at the equator (Hijmans et al., 2005). To deal with collinearity, environmental variables were screened using the SDMTune v1.1.5 (Vignali et al., 2020) package in R (R Core Team, 2013). The variables having a correlation coefficient with an absolute value >0.7 (Figure S1) were removed, as suggested by Dormann et al. (2013). Variance inflation factor (VIF) measures the proportion of a regressor's variability that is explained by the other regressors in the model as a result of their correlation (Craney & Surles, 2002). We then calculated VIF values for the remaining variables using the *vifstep* function of the usdm package, and removed any variables with a VIF > 5. The final variables used to perform SDM included four bioclimate variables, bio3 (isothermality), bio4 (temperature seasonality), bio8 (mean temperature of wettest quarter) and bio12 (annual precipitation).

We also downloaded bioclimate variables from WorldClim for future climate conditions in the 2050s (the average of period 2041–2060) and 2070s (the average of period 2061–2080), using the same resolution and the same bioclimatic layers used in the current conditions (bio3, bio4, bio8, bio12). We included two representative concentration pathways (RCP) scenarios, RCP 2.6 and RCP8.5, based on the CCSM4 (the community climate system model, version 4) climate model, with RCP2.6 and RCP 8.5 representing low (optimistic) and high (pessimistic) greenhouse gas emission scenarios, respectively. The CCSM4 model has been effectively utilized in China to predict how climate change will impact the distribution of plant species (Zhao et al., 2021). Paleoclimate data for the LGM were also downloaded from the WorldClim 1.4 dataset.

### 2.3 Species distribution modelling and evaluation

Biomod2 v4.1.2 (Thuiller et al., 2022) was used to model species distributions under the current climatic scenario, and to project for the LGM and the future in the 2050s and the 2070s. Four commonly used algorithms were applied in this study: general linear models (GLM), general boosted regression trees models (GBM, also called BRT), random forests (RF) and maximum entropy (MaxEnt) (Brauer et al., 2023). The maximum number of iterations was set as 1000 for MaxEnt, GBM was developed with a maximum number of trees set to 2000, and RF was fitted with 1000 trees. 80% of the occurrences were randomly selected for training the models and the remaining 20% for evaluating. Pseudo‐absence points were randomly generated with the number equal to three times of true‐presence records as suggested by the authors of Biomod2 (Thuiller et al., 2022). The number of replicates was set as 5 to calibration/validation models, and the number of permutations was set as 5 to estimate variable importance. A total of 20 different models (4 algorithms × 5 cross‐validation runs) were established for each species.

ROC (the area under the receiver operating characteristics curve), kappa (Cohen's kappa statistic) and the TSS (true skill statistic) were used to measure model performance. ROC values vary from 0 to 1, with values close to 1 indicating that the model result is accurate. Both the kappa statistic and TSS statistic range from −1 to +1, where kappa = 1 indicates perfect agreement and −1 indicates 100% of prediction does not agree with the truth (Allouche et al., 2006), TSS = 1 indicates a perfect performance, TSS = 0 or −1 indicates no better than random performance (Cohen, 1960). Only the models with kappa >0.8, TSS > 0.8, and ROC >0.9 were used to build an ensemble model with a weighted mean approach.

### 2.4 Change in size of potential ranges

Based on the TSS threshold, continuous suitability distribution maps were reclassified to binary maps (absence/presence, 0/1). The *BIOMOD_RangeSize* function in Biomod2 was then used to analyse the potential changes in the distribution of suitable habitat and range size changes for the two maple species studied, including the areas predicted to be lost (‘lost’), the areas currently suitable and predicted to remain suitable in the future (‘stable’), the areas currently unsuitable and predicted to become suitable in the future (‘gain’), the percentage of currently suitable areas predicted to be lost (‘PercLost’), the percentage of new areas considering the species' current range size (‘PercGain’) and the total predicted change in range size of suitable habitat between current and future conditions (‘RangeChange’).

## 3 RESULTS

### 3.1 Importance of variables and model performance

**FIGURE 2:**
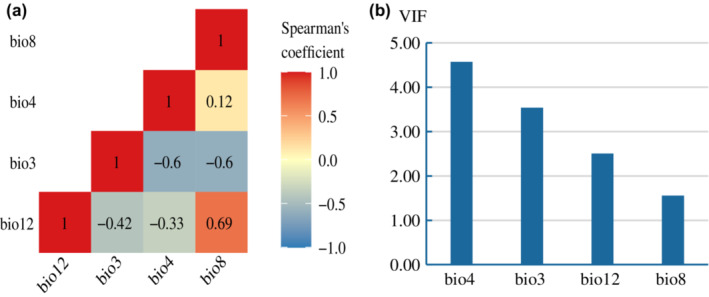
Collinearity among bioclimatic variables. (a) Spearman's correlation coefficients; (b) Variance inflation factor (VIF).

All variables used for the modelling of species distributions had correlation coefficients with absolute values <0.7, and VIF < 5, indicating weak collinearity (Figure 2). The mean ROC values of the ensemble models were 0.99 and 0.98, the mean TSS values were 0.92 and 0.88 and the mean kappa values were 0.87 and 0.84 for *A*. *caesium* and *A*. *stachyophyllum*, respectively (Table 1), indicating robust prediction and good predictive accuracy.

**TABLE 1:** Model performance and variable importance of ensemble models.

Abbreviation	Bioclimatic variables	Performance threshold	*Acer caesium*	*Acer stachyophyllum*
ROC		Area under the receiver operating characteristics curve	0.99	0.98
TSS		True skill statistic	0.92	0.88
kappa		Cohen's kappa statistic	0.87	0.84
bio4	Temperature seasonality		62.91%	53.76%
bio12	Annual precipitation		20.76%	26.28%
bio8	Mean temperature of wettest quarter		18.49%	26.06%
bio3	Isothermality		0.72%	2.44%

Temperature seasonality (bio4) was the most important bioclimatic variable, contributing 62.91% and 53.76% to the potential distributions of *A*. *caesium* and *A*. *stachyophyllum*, respectively (Table 1). Annual precipitation (bio12) was the second most important bioclimatic variable, accounting for 20.76% and 26.28% of the potential distributions of *A*. *caesium* and *A*. *stachyophyllum*, respectively. Mean temperature of the wettest quarter (bio8) was also important, contributing 18.49% and 26.06% to the potential distributions of *A*. *caesium* and *A*. *stachyophyllum*, respectively.

### 3.2 Predicted distribution patterns

**FIGURE 3:**
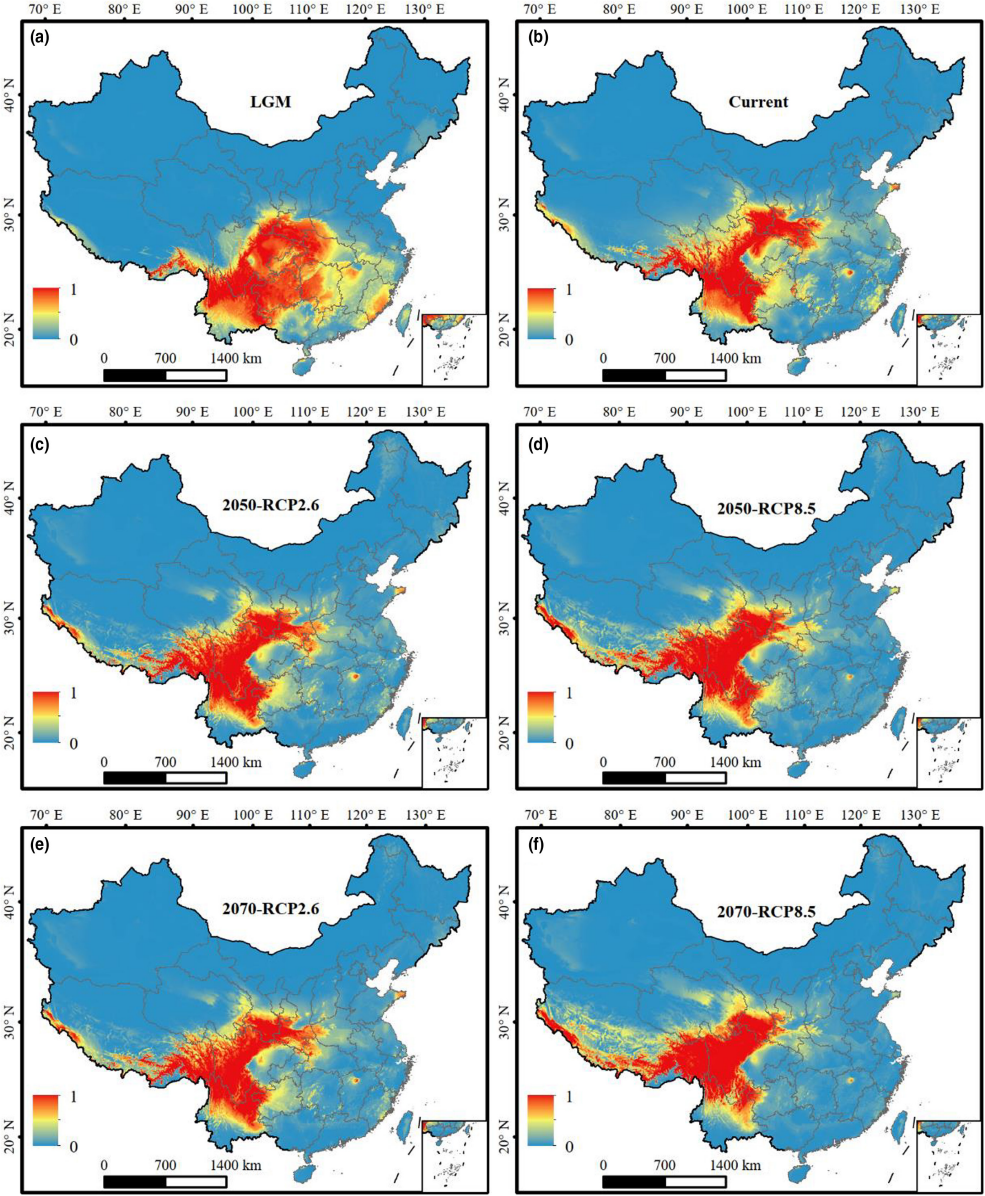
Predicted distributions of *Acer caesium* in China (a) during the LGM, (b) under current climatic scenario and (c, d) in the 2050s and (e, f) in the 2070s under two future climatic scenarios, RCP2.6 and RCP8.5. LGM, the Last Glacial Maximum; RCP2.6 and RCP8.5, two representative concentration pathway scenarios, with RCP2.6 representing an optimistic scenario and RCP8.5 representing a pessimistic scenario; 0, representing maximum unsuitability; 1, representing maximum suitability.

**FIGURE 4:**
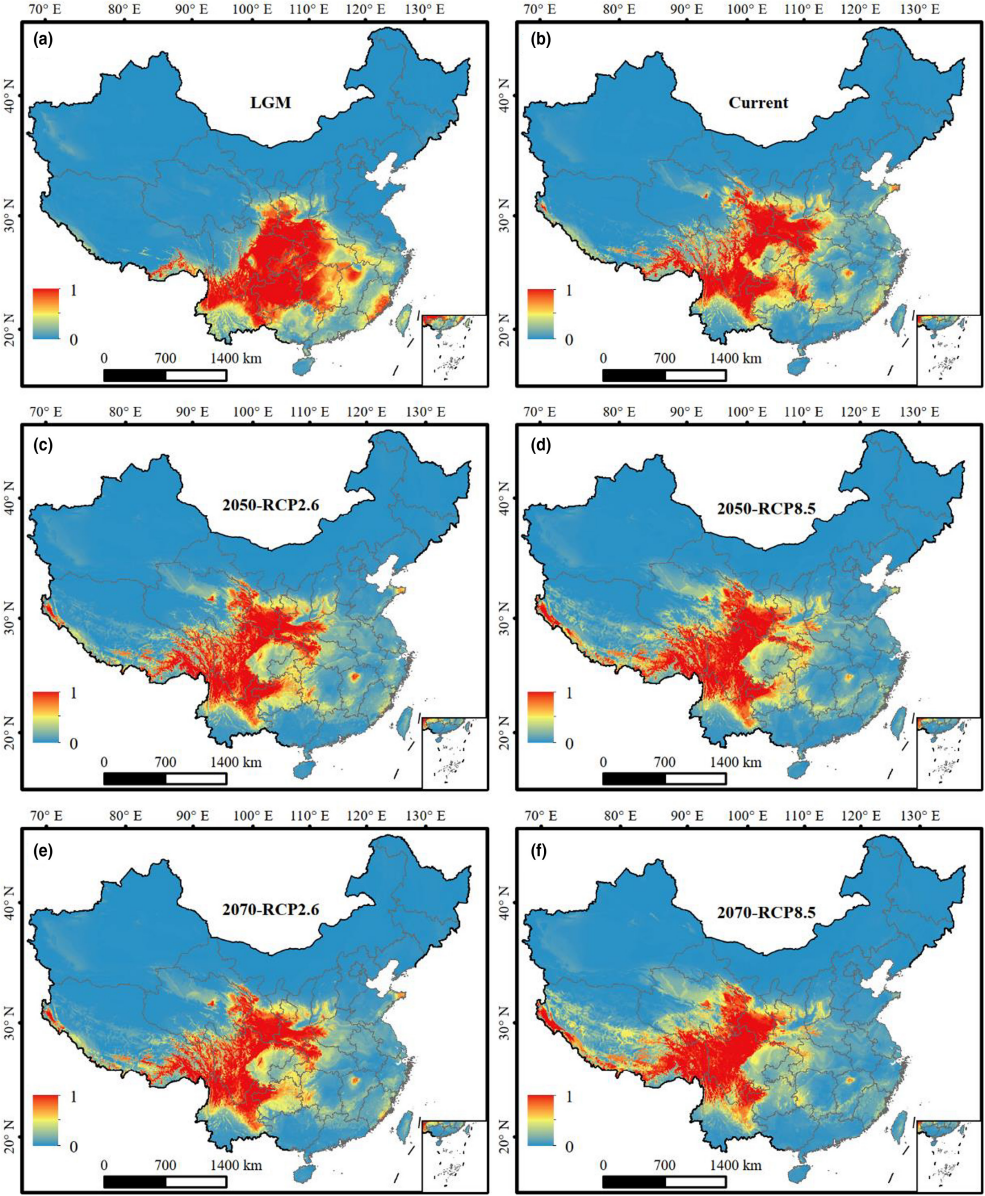
Predicted distributions of *Acer stachyophyllum* in China (a) during the LGM, (b) under current climatic scenario and (c, d) in the 2050s and (e, f) in the 2070s under two future climatic scenarios, RCP2.6 and RCP8.5. LGM, the Last Glacial Maximum; RCP2.6 and RCP8.5, two representative concentration pathway scenarios, with RCP2.6 representing an optimistic scenario and RCP8.5 representing a pessimistic scenario; 0, representing maximum unsuitability; 1, representing maximum suitability.

At the time of the LGM, potentially highly suitable habitats for *A*. *caesium* were concentrated in eastern, northeastern and northwestern Yunnan, southeastern Tibet, southeastern and eastern Sichuan, southern Gansu and southern Shaanxi, western Hubei, western Hunan, Chongqing, Guizhou Provinces (Figure 3a). Under the current climatic scenario, the areas of potentially highly suitable habitat for *A*. *caesium* will decrease from that predicted during the LGM in the eastern regions in Chongqing, Guizhou, Hubei, Hunan, eastern Sichuan, western Guangxi and Middle Yunnan Provinces and expand in southern Gansu, western Sichuan and southeastern Tibet Provinces (Figure 3b). Under the RCP2.6 scenario predicted for the 2050s, potentially suitable habitat for *A*. *caesium* continued to shrink in the eastern regions and showed an expansion towards the west (Figure 3c). This trend was predicted to be stronger under the RCP8.5 scenario in the 2050s (Figure 3d). Similar patterns were also predicted under the RCP2.6 and RCP8.5 scenarios for the 2070s (Figure 3e,f). Overall, the predicted potentially suitable habitat for *A*. *caesium* showed a trend of contraction in the east and expansion towards the west from the LGM and into the future. The results of the SDMs for *A*. *stachyophyllum* revealed similar patterns and dynamics to those for *A*. *caseum* (Figure 4a–f).

### 3.3 Distributional responses

**FIGURE 5:**
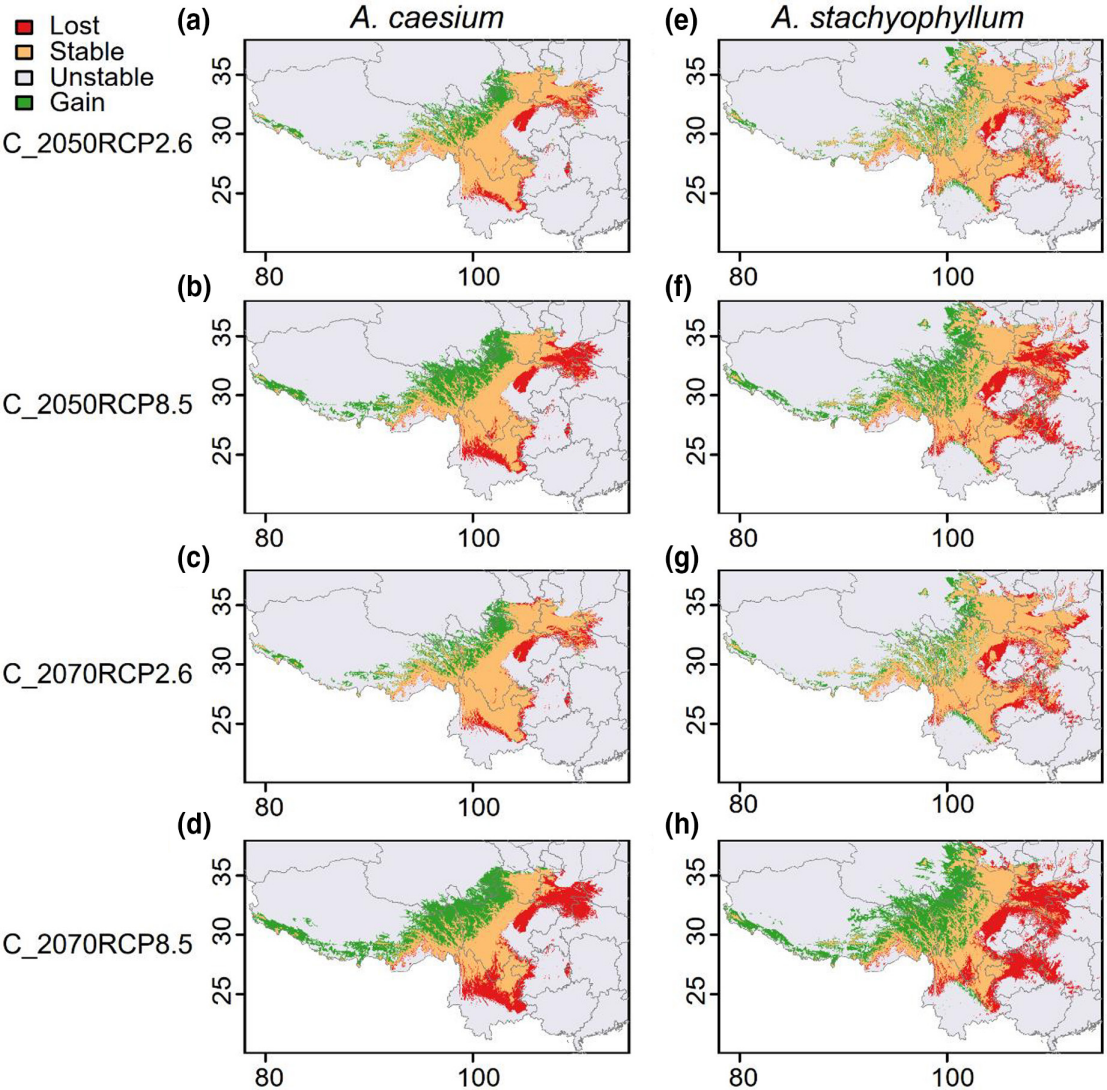
Spatial changes in the predicted distribution ranges of the two maple species between the present and the future (2050s and 2070s). (a, e) C‐2050RCP2.6, changes between current and the RCP2.6 scenario in the 2050s; (b, f) C‐2050RCP8.5, changes between current and the RCP8.5 scenario in the 2050s; (c, g) C‐2070RCP2.6, changes between current and the RCP2.6 scenario in the 2070s; (d, h) C‐2070RCP8.5, changes between current and the RCP8.5 scenario in the 2070s; lost, areas currently suitable but potentially unsuitable in the future; stable, areas currently suitable and likely to remain suitable in the future; unsuitable, areas unsuitable at present and remaining unsuitable in the future; gain, areas currently unsuitable but potentially suitable in the future.

The result of spatial changes indicated that the two maple species might have shifted their distributions towards the west of their current distribution ranges from the LGM to the present scenario and that they will continue to shift their distributions towards the west of their current distribution ranges in response to climate change. This trend was more evident under the pessimistic scenarios (RCP8.5) than that under the optimistic scenarios (RCP2.6), and it was more evident in the 2070s than in the 2050s (Figure 5). These two maple species are predicted to gain suitable habitat in the southeastern parts of the QTP. The predicted potential suitable habitat areas under the current climatic conditions in southeastern Tibet, the Hengduan Mountains in northwestern Yunnan and western Sichuan, the Qinling‐Daba Mountains in southern Gansu and the Wumeng‐Daliang Mountains in northeastern Yunnan, western Guizhou and southeastern Sichuan, were predicted to remain stable in the 2050s and the 2070s (Figure 5).

The range sizes of suitable areas for *A*. *caesium* are predicted to increase by 17.42%–24.37% under all predicted scenarios in the future (2050s and 2070s, Table 2), which are evidently in the western parts of their current range (Figure 5). The range sizes of suitable areas for *A*. *stachyophyllum* are predicted to have an average of 0.47%–0.73% increase under the predicted scenarios in the 2050s, while an average of 1.06%–2.01% decrease in the 2070s (Table 2). The potential suitable habitat areas of *A*. *caesium* and *A*. *stachyophyllum* are all predicted to shift towards the eastern regions of the QTP, which is more evident under the RCP8.5 scenario (Figure 5). The eastern and southern parts of currently suitable areas are predicted to be lost in the future, both in the 2050s and in the 2070s (Figure 5). The most significant range size change, contraction or expansion, are predicted under high emission scenarios in the 2070s (Figure 5 and Table 2), with 35.74% and 45.71% of current suitable habitat area lost and 57.98% and 44.65% of suitable habitat area gain for *A*. *caesium* and *A*. *stachyophyllum*, respectively, under RCP8.5 scenarios in the 2070s (Table 2).

**TABLE 2:** Changes in potentially suitable habitat areas for the two maple species under two future climatic scenarios, RCP2.6 and RCP8.5, in the 2050s and 2070s.

Species	Scenario	Area lost (×10^5^ km^2^)	Area stable (×10^5^ km^2^)	Area gained (×10^5^ km^2^)	PercLost (%)	PercGain (%)	RangeChange (%)
*Acer caesium*	2050RCP2.6	1.41	8.80	3.25	13.78	32.68	18.91
	2050RCP8.5	2.47	7.74	4.87	24.23	48.60	24.37
	2070RCP2.6	1.46	8.75	3.16	14.27	31.69	17.42
	2070RCP8.5	3.64	6.58	5.83	35.74	57.98	22.24
*Acer stachyophyllum*	2050RCP2.6	2.98	12.58	3.08	19.18	19.91	0.73
	2050RCP8.5	5.15	10.40	5.21	33.14	33.61	0.47
	2070RCP2.6	3.24	12.31	2.93	20.89	18.88	−2.01
	2070RCP8.5	7.10	8.45	6.93	45.71	44.65	−1.06

*Note*: Potentially suitable habitat changes were estimated in Biomod2. Negative values represent reduction and positive values represent expansion in the future.

## 4 DISCUSSION

In this study, we chose two widespread maple species and projected their past (LGM), present and future (2050s and 2070s) potential distributions using species distribution modelling implemented in Biomod2 based on occurrence data and bioclimatic variables. The results showed that of the four environmental variables used for modelling in this study, the most important environmental variables in predicting habitat suitability for the two maple species were temperature seasonality (bio4) and annual precipitation (bio12), with temperature (bio4) being more important than precipitation (bio12). Similar findings have been reported in explaining richness patterns in southern China for the genera *Rhododendron* (Shrestha et al., 2018) and *Viburnum* (Lyu et al., 2022), and for the potential distribution of another maple (*A*. *monspessulanum*; Aouinti et al., 2022). Our results contrast with those of Liu, Yang et al. (2022), who found that annual precipitation was the most important variable in predicting habitat suitability for 15 of 20 threatened maple species. Stable climatic conditions are important for the survival and dispersal of taxa, while extreme weather events may have particularly strong impact on species. *A*. *caesium* and *A*. *stachyophyllum* are distributed in the montane regions of the QTP and the Hengduan Mountains. And the effect of temperature seasonality is more pronounced in regions of high topographic relief, especially in southern China with a monsoon climate (Shrestha et al., 2018).

Precipitation (bio12) also had an important affect on the distribution of *A*. *caesium* and *A*. *stachyophyllum* (20.76%, 26.28%). Mean temperature of wettest quarter (bio8) was the third important factor, which contributed 18.49% and 26.06% to the habitat suitability of *A*. *caesium* and *A*. *stachyophyllum*, respectively. This might be explained by the defoliation traits of maple species in the driest quarter and the monsoon climate (the wet and dry seasons are distinct) in southern China. Germination of maple species begins with the onset of the rainy season. In China, the wettest quarter, which lasts from July to September, is when maple trees experience their most active development and fruit production. During this period, temperature is therefore an important factor in their growth.

Several factors were not considered in our study, including soil type, land use, landscape features and the dispersal potential and physiological tolerance of the studied species, as these data are not available for the LGM period. These factors might affect the dispersal of these species and therefore determine whether they will be able to track global change. This is particularly true in southern China, where a variety of mountains and deep gorges intersect to create many topographies.

The predicted distributions of potentially suitable habitat for *A*. *caesium* and *A*. *stachyophyllum* were predicted to be larger during the LGM than they are currently, suggesting that these maple species greatly expanded their ranges in the QTP and the Hengduan Mountains during the LGM. Previous findings based on nuclear data and SDM also suggested that some conifers might have expanded their distributional ranges during the LGM (Liu et al., 2014). Abundant well‐preserved fossils of maple species have recently been found in the Oligocene Shangganchaigou Formation from the northern parts of the QTP, which also indicated that the historical distribution of maple species in the Oligocene (~34–23 Ma) was wider than today (Yang, 2018).

The regions predicted to be highly suitable for all two maple species under the current climate scenario were concentrated in southwestern China, where there are complex habitat mosaics of mountainous topography and long‐term stable climate (Tang et al., 2018). While the low‐altitude regions, particularly in the Sichuan Basin and Chongqing Province were predicted to be unsuitable under the current climate scenario compared to that of the LGM period, temperature may be responsible for this distribution pattern. The temperature during the LGM period was 4.0 ± 0.8°C lower than present (Annan & Hargreaves, 2013). In fact, the actual distribution of *A*. *caesium* and *A*. *stachyophyllum* is mainly in mountain areas (2300–3200 m) in the wild. It is feasible that a cold‐temperate environment is more favourable for these two maple species. We, therefore, infer that the cold climate during the LGM created favourable conditions for some cold‐tolerant maple species to expand their range. Subsequently, the glaciers retreated and the Earth gradually warmed, particularly at low altitudes. The warmer climate jeopardized the habitat of maple species and threatened their survival, particularly in low‐elevation regions. These species are now restricted to mountain regions.

The predicted changes in distribution patterns under the RCP2.6 and RCP8.5 scenarios were not identical for the two species. This could be explained by the fact that the mountains in the Hengduan Mountains and the QTP have different orientations, resulting in differing distribution patterns in the region. A great expansion could be observed in the southeastern QTP and the Hengduan Mountains under the RCP8.5 scenario in both the 2050s and 2070s, while significant contractions could be seen in southern Yunnan, Hubei, Hunan and eastern Sichuan Provinces under the RCP8.5 scenario in both the 2050s and 2070s. In particular, the potential distributions of habitat suitable for each of the two maple species will move towards the west, especially towards the southeastern QTP regions and the Hengduan Mountains, which is consistent with earlier research (Gao et al., 2020; Li et al., 2019; Xu, 1996, 1998). Similar patterns have been reported for the genera *Cyanathus* and *Primula* (He et al., 2019), and five dominant *Abies* species in southwestern China (Liao et al., 2020). Our results show that some areas in southeastern Tibet, the Hengduan Mountains, the Qinling‐Daba Mountains and the Wumeng‐Daliang Mountains, are potentially suitable for these two maple species under current climate condition and will remain suitable under predicted future climate change conditions. This may be explained by the possibility that the complex topography of the mountains in southwestern China may have provided more available surface area as a result of upslope movement, which may have contributed significantly to the increase in range size of some montane species under climate change (Liang et al., 2018). Previous work showed that these areas are not covered by the existing distribution of nature reserves, it is therefore urgent to incorporate these areas into protection areas in conservation planning (Hua et al., 2022).

Our findings imply that widespread plant species may also be vulnerable to climate change, as the ranges of the two widespread maple species were all predicted to shift significantly under both pessimistic and optimistic climate change scenarios. Furthermore, the two maple species in our study could potentially spread towards the QTP in the future. This suggests that other species, not just those threatened species currently native to the QTP, but also those categorized as Least Concern (LC) and those not native to the QTP, should be considered in conservation studies when designing nature reserves and national parks in the future. While migration of plant species into new suitable areas could potentially be assisted by artificial measures such as *ex situ* conservation, reintroductions, botanical garden collections and plantations. It is, therefore, necessary to predict the responses of plant species to global change in advance using SDM, which could provide an important basis for biodiversity conservation and will be critical in informing future conservation actions.

## AUTHOR CONTRIBUTIONS

**De Tuan Liu:** Formal analysis (equal); writing – original draft (equal). **Jian Ying Chen:** Data curation (equal); resources (equal); writing – review and editing (equal). **Wei Bang Sun:** Conceptualization (equal); funding acquisition (equal); project administration (equal); supervision (equal); writing – review and editing (equal).

## FUNDING INFORMATION

This work was supported by the Second Tibetan Plateau Scientific Expedition and Research Program (2019QZKK0502), by the Key Basic Research Program of Yunnan Province, China (202101BC070003) and by the Science and Technology Basic Resources Investigation Program of China (2017FY100100).

## CONFLICT OF INTEREST STATEMENT

The authors declare that they have no known competing financial interests or personal relationships that could have appeared to influence the work reported in this paper.

## Supporting information


Table S1.

## Data Availability

The data that support the findings of this study are openly available in figshare at 10.6084/m9.figshare.22893317.
