# Supplementary material for: Distributional responses to climate change of two maple species in southern China
Source: Ecol Evol. 2023 Aug 31;13(9):e10490. doi: 10.1002/ece3.10490 (PMC10468973; doi:10.1002/ece3.10490)
Supplement: Supplementary file 1 — Table S1. [file ECE3-13-e10490-s001.docx]

**Appendix. A**

**Table S1 The 19 Bioclimatic variables downloaded from WorldClim.**

| **Abbreviation** | **Bioclimatic variables** |
| --- | --- |
| bio1 | Annual Mean Temperature |
| bio2 | Mean Diurnal Range (Mean of monthly (max temp - min temp)) |
| bio3 | Isothermality (bio2/bio7) (×100) |
| bio4 | Temperature Seasonality (standard deviation ×100) |
| bio5 | Max Temperature of Warmest Month |
| bio6 | Min Temperature of Coldest Month |
| bio7 | Temperature Annual Range (bio5-bio6) |
| bio8 | Mean Temperature of Wettest Quarter |
| bio9 | Mean Temperature of Driest Quarter |
| bio10 | Mean Temperature of Warmest Quarter |
| bio11 | Mean Temperature of Coldest Quarter |
| bio12 | Annual Precipitation |
| bio13 | Precipitation of Wettest Month |
| bio14 | Precipitation of Driest Month |
| bio15 | Precipitation Seasonality (Coefficient of Variation) |
| bio16 | Precipitation of Wettest Quarter |
| bio17 | Precipitation of Driest Quarter |
| bio18 | Precipitation of Warmest Quarter |
| bio19 | Precipitation of Coldest Quarter |


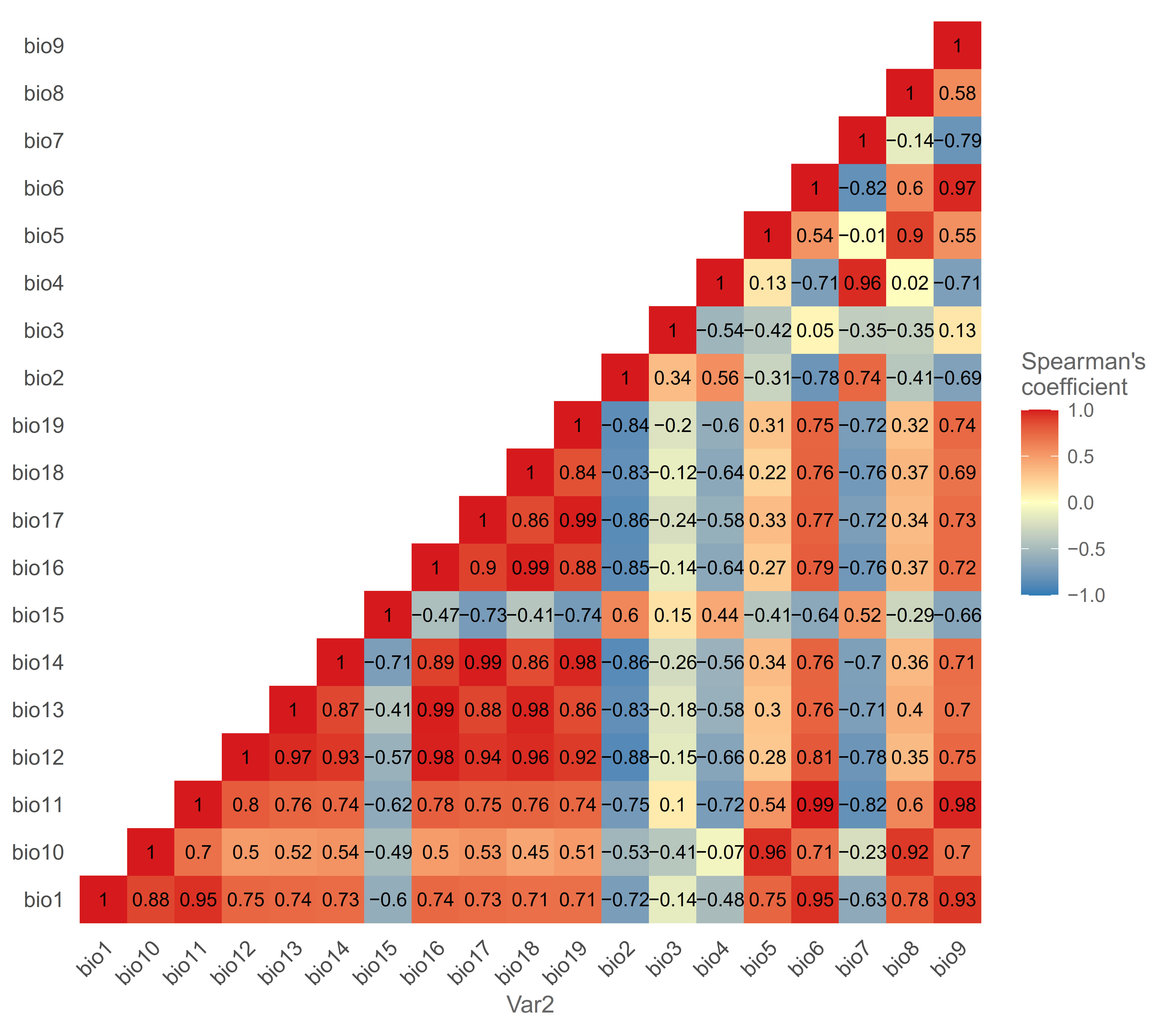


**Figure S1 Spearman’s correlation coefficients among climatic variables.**
